# Iron-enriched diet contributes to early onset of osteoporotic phenotype in a mouse model of hereditary hemochromatosis

**DOI:** 10.1371/journal.pone.0207441

**Published:** 2018-11-14

**Authors:** Márcio Simão, António Camacho, Agnès Ostertag, Martine Cohen-Solal, I. Jorge Pinto, Graça Porto, Ea Hang Korng, M. Leonor Cancela

**Affiliations:** 1 PhD Program in Biomedical Sciences, Department of Biomedical Sciences and Medicine (DCBM), University of Algarve, Faro, Portugal; 2 Centre of Marine Sciences (CCMAR), University of Algarve, Faro, Portugal; 3 Department of Orthopedics, Hospital de Cascais, Alcabideche, Portugal; 4 Inserm U1132, Bioscar, Hôpital Lariboisiére, pôle locomoteur, service de rhumatologie, Université Paris 7 Denis Diderot, Paris, France; 5 Basic and Clinical Research on Iron Biology, Institute for Molecular and Cell Biology (IBMC) and I3S –Instituto de Investigação e Inovação em Saúde, University of Porto, Porto, Portugal; 6 Pathology and Molecular Immunology Department, Institute of Biomedical Sciences Abel Salazar (ICBAS), University of Porto, Porto, Portugal; 7 Hematology Service, Hospital de Santo António, Centro Hospitalar do Porto, Porto, Portugal; 8 Department of Biomedical Sciences and Medicine (DCBM), University of Algarve, Faro, Portugal; 9 Algarve Biomedical Center, University of Algarve, Campus de Gambelas, Faro, Portugal; Lady Davis Institute for Medical Research, CANADA

## Abstract

Osteoporosis is associated with chronic iron overload secondary to hereditary hemochromatosis (HH), but the causative mechanisms are incompletely understood. The main objective of this study was to investigate the role of dietary iron on osteoporosis, using as biological model the Hfe-KO mice, which have a systemic iron overload. We showed that these mice show an increased susceptibility for developing a bone loss phenotype compared to WT mice, which can be exacerbated by an iron rich diet. The dietary iron overload caused an increase in inflammation and iron incorporation within the trabecular bone in both WT and Hfe-KO mice. However, the osteoporotic phenotype was only evident in Hfe-KO mice fed the iron-enriched diet. This appeared to result from an imbalance between bone formation and bone resorption driven by iron toxicity associated to Hfe-KO and confirmed by a decrease in bone microarchitecture parameters (identified by micro-CT) and osteoblast number. These findings were supported by the observed downregulation of bone metabolism markers and upregulation of ferritin heavy polypeptide 1 (*Fth1*) and transferrin receptor-1 (*Tfrc*), which are associated with iron toxicity and bone loss phenotype. In WT mice the iron rich diet was not enough to promote a bone loss phenotype, essentially due to the concomitant depression of bone resorption observed in those animals. In conclusion the dietary challenge influences the development of osteoporosis in the HH mice model thus suggesting that the iron content in the diet may influence the osteoporotic phenotype in systemic iron overload conditions.

## Introduction

Iron is one of the most important minerals in biological systems, with relevant roles in oxygen transport, energy production, DNA synthesis and regulation of several enzymes [1–3]. When in excess, iron contributes to the development of several diseases such as liver cirrhosis, hepatocarcinoma, cardiac cirrhosis, diabetes mellitus, osteoarthritis and osteoporosis [4,5]. Hereditary hemochromatosis (HH) is a genetic disorder characterized by systemic iron overload due to hepcidin (HAMP) deficiency [3,5]. The majority of HH cases (~80%) have been associated with mutations in Human Hemochromatosis protein (*HFE)* gene, essentially C282Y, with a prevalence of 1:200 to 1:300 in populations with northern European ancestry [4,6]. HFE can regulate intracellular iron entry by competing with Transferrin (TF) for Transferrin Receptor 1 (TFRC) binding and when mutated it affects HAMP expression negatively [4,6].

Osteoporosis (OP) has been associated to HH and iron overload [7–10]. A significant percentage of HH patients develop low bone mass (74–79%), eventually resulting in OP (25–34%) that worsens with age [9,10]. HH-related OP results from the imbalance between bone formation and resorption leading to deterioration of bone microarchitecture and increased fracture risk [2,9,11]. OP onset and progression are subject to synergistic factors, such as a decrease in levels of estrogen and progesterone in postmenopausal women, associated with iron overload and oxidative stress [2,11]. Nutrition is also an important variable in OP pathophysiology since a disruption of the nutritional equilibrium of several essential minerals, like Cu, Fe, Mg and Zn, as well as iron overload associated with diet and age, have been described as factors that can contribute to OP [8,12,13].

A preliminary evaluation of bone microarchitecture in a murine model of HH performed in our laboratory showed incomplete penetration of bone loss phenotype (Fig A in S1 File), suggesting that other variables could contribute to the development of HH-related osteoporosis. Therefore, the aim of this study was to determine if, by providing a supplementary dietary iron intake, we could promote the development of an osteoporotic phenotype.

## Material and methods

All chemical reagents described in this section were obtained from Sigma-Aldrich unless stated otherwise.

### Ethical statement

All the individuals involved in animal handling and experimentation received proper training (category B courses accredited by FELASA, the Federation of Laboratory Animal Science Associations). All experimental procedures involving animals followed the European Directive 2010/63/EU and the related guidelines (European Commission, 2014). The animal experiments were performed on the animal facility of the Institute for Molecular and Cell Biology (IBMC), University of Porto, Portugal, which was licensed by Portuguese National Authority for Animal Health (DGAV) on October 23^rd^ of 1999, according with articles 27^th^ and 36^th^ of ministerial decreed n° 1005/92 of Portuguese legislation (Decreto-Lei 113/2013) for animal experimentation and welfare. In addition, experiments were authorized by the Institute for Molecular and Cell Biology's animal ethics committee.

### Biological model

The murine model of HH used in this study, Hfe-KO in a C57BL/6 background, was described by Bahram et al. (1999) [14] and backcrossed with C57BL/6 mice colony at IBMC. The Hfe-KO mice are homozygotes for a removal of exons 2 and 3 of the HFE gene, leading to loss of function of the HFE protein [14,15]. All mice were maintained at the IBMC animal facility kept in a 12hrs light/dark cycle, in specific pathogen-free conditions, and had *ad libitum* access to water and food (standard diet-Harlan 2018S, Teklad Global Rodent Diets, iron content 200 mg/kg) from the time of weaning until sacrifice.

### Experimental design

A total of 24 male mice, 12 Hfe-KO and 12 WT, were used in this study. Since weaning and until the completion of 3 months of age the animals were fed with the standard diet. Between 3 and 6 months of age, 6 animals of each strain were fed with an iron-enriched diet (Harlan 2018S supplemented with 1% of iron carbonyl) while the remaining animals were kept on the standard diet until they completed 6 months of age. At this time the mice were anesthetized with a combination of ketamine (1mg/10g body weight, i.p., Merial), xylazine (0.1mg/10g body weight, i.p., Bayer) and acepromazine (0.03mg/10g body weight, i.p., Vétoquinol) and sacrificed. The hindlimbs were collected and prepared for the different analyses, the right hindlimb for histological, histomorphometric and micro-CT analysis and the left hindlimb for total RNA isolation.

One animal from WT group under control diet (C) died and was excluded from this study because it showed signs of fragility and disease which compromised the animal welfare. After animal identification, it was anesthetized accordingly with described previously and sacrificed by cervical dislocation. The final distribution of animals by groups was the following: Hfe-KO with control diet (KO): 6 mice, Hfe-KO with iron-enriched diet (KO+I): 6 mice; WT with control diet (C): 5 mice; WT with iron-enriched diet (C+I): 6 mice.

### Quantification of iron biochemical parameters in serum

Blood was collected by intra-cardiac puncture to tubes containing Heparin-Lithium and sent to an external certified commercial laboratory (DNAtech, Lisbon, Portugal) for determination of serum iron, serum ferritin and serum transferrin saturation similar to what was described previously [16].

### Micro computed tomography (micro-CT)

Bone microarchitecture was always assessed using the right hindlimb of each mouse. The samples were fixed for 24h with 4% paraformaldehyde (PFA) in phosphate-buffered saline (PBS) at pH7.4 and preserved in 70% ethanol until data acquisition by micro-CT with a Skyscan 1272 X-ray computed microtomography (Bruker, Belgium). Samples were wrapped in laboratory film (Parafilm), embedded in distilled water and placed inside 2mL tubes (Sarstedt). For image acquisition, the following parameters were used: X-ray tube potential 70 kV, X-ray tube current 142 μA, 0.5mm Al filter, rotation step 0.4°, isotropic voxel size 5 μm^3^ and exposure time 1500ms. For data reconstruction, the NRecon software (v1.6.9.8, Bruker, Belgium) was used, with Gaussian smoothing, ring artefact correction and 40% beam hardening correction applied. Using Dataviewer software (v1.4.4, Bruker, Belgium) each dataset was normalized regarding its orientation and saved in trans axial (X-Y) projections, and then exported to CTAn software (v1.13.11.0, Bruker, Belgium). To measure bone microarchitecture parameters for each dataset, growth plate plus 0.25mm was used as structural reference between each sample and then, using CTAn software, the regions of interest (ROI) for 1.2mm in length were selected and three-dimensional microarchitecture parameters calculated for both the femur and tibia and expressed according with the ASBMR recommendations [17]. Microarchitecture parameters included bone volume fraction (BV/TV), bone volume (BV), trabecular thickness (Tb.Th), trabecular number (Tb.N), trabecular separation (Tb.Sp), and structural model index (SMI), indicative of trabecular organization [18]. Three-dimensional reconstructions representative of bone status evaluation in femur and tibia were done with CTvox software (v3.1.1, Bruker, Belgium).

### Histomorphometric analysis

After micro-CT analysis, femurs were included in methyl methacrylate (MMA) at 4°C. Serial 5μm sections were cut in three distinct levels, eight sections per level, with a 50μm interval between levels. Relative osteoid surface (Osteoid / Bone surfaces = OS/BS *100%) and number of osteoblast per bone surface N.Ob/BS (mm^-1^) were determined in 2 sections per level in each animal by staining with toluidine blue (1%) as described previously [19]. The Axioimager Z2 microscope (Zeiss, Germany) was used for osteoid and bone surfaces identification and each area was measured with Axiovision software (Zeiss, Germany) at 200x magnification. Bone and osteoid surfaces were measured in the distal metaphysis of the femur. To determine the osteoblasts number (N.Ob) in OS a magnification of 400x was used (Axioimager Z2 microscope, Zeiss, Germany). Osteoclasts were stained with naphthol AS-TR (3- hydroxy-2-naphthoic acid 4-chloro-2-methylanilide) phosphate for tartrate-resistant acid phosphatase (TRAP) detection and counterstained with Toluidine blue (0.5%), [20]. Osteoclasts were counted and expressed relatively to bone surface as Oc.S/B.Ar (mm^2^). To evaluate total iron accumulation in femur trabecular bone, sections were stained with Perl’s solution ((2% Ferrocyanide potassium) 1:1 (2% HCl (37%)) and counterstained with Nuclear Fast Red (1%).

### Expression of bone tissue markers in tibia

RNA extraction of bones derived from the left hindlimb of both WT and Hfe-KO mice for each condition tested (C, C+I, KO and KO+I), was done with Isol-RNA Lysis Reagent (5 Prime-VWR) according with the manufacture instructions, followed by RNA purification with GeneJET RNA purification Kit (Thermo-Scientific). RNA samples were quantified and assessed for purity using a Nanodrop 2000 (Thermo Scientific). RNA integrity was evaluated by Experion system (Bio-Rad) and then 1μg of RNA for each condition was submitted to DNase treatment (Promega) and reverse transcribed using 1μL of oligo(dT) adapter primer (10μM) and 1μL of M-MLV Reverse Transcriptase (1U/μL, Invitrogen), following the manufacture instructions. The real-time polymerase chain reaction (qPCR), (20μl) was performed using as template 2μl of 1:10 cDNA dilution for each condition tested, 10μl of SsoFast Evagreen Taq supermix (BioRad), 0.6 μl of primers for target genes or housekeeping genes (300nM), (Table A in S1 File) and the final volume completed with purified water free from RNases (Sigma). Amplification by qPCR was performed in a CFX96 System (Bio-Rad) in three independent qPCR reactions for each sample. To determine the levels of gene expression all results were normalized to levels of expression of Glyceraldehyde-3-Phosphate Dehydrogenase (*Gapdh*) and relative expression was determined by the ΔΔCt method[21] using the condition C (WT with standard diet) as control group for basal expression (1).

### Statistical analysis

The results are expressed as group means and associated mean standard error (SEM). Each group was evaluated for normality with Kolmogorov-Smirnov test and confirmed for application of parametric analysis. Analysis for the impact of iron rich diet on serum iron parameters, bone microarchitecture, bone histomorphometric parameters and gene expression were done by comparison with T-test (Welch’s correction) between control diet and iron rich diet groups within each genotype, (C vs C+I and KO vs KO+I) with p<0.05 for statistical significance. The correlation between Trap positive cells/bone surface and bone microarchitecture parameters was done by Pearson method with p<0.05 for significance.

## Results

### Increased systemic and bone iron accumulation in Hfe-KO mice

**Fig 1:**
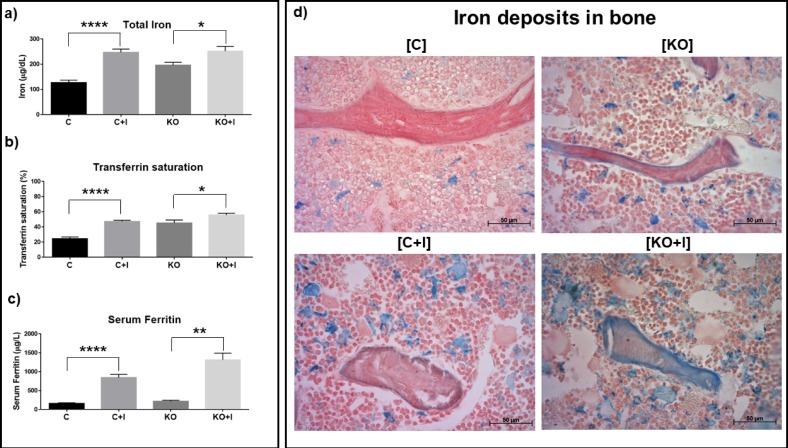
Evaluation of systemic iron accumulation in blood serum and bone. In blood serum: a) Total iron concentration; b) Transferrin saturation; c) Total concentration of serum ferritin. In bone: d) Detection of iron deposits (stained in blue) in femur bone trabeculae and bone marrow through Perls staining. Each group analyzed was composed by n = 6 animals for [C+I], [KO] and [KO+I], and by n = 5 for [C]. Statistical comparison was performed with T-test (Welch’s correction) between control and iron-enriched diets for both WT ([C] and [C+I]]) and Hfe-KO mice ([KO] and [KO+I])). Group deviations are expressed as standard error to the mean (SEM). Statistical significance: (*)-p<0.05; (**)-p<0.01; (****)-p<0.0001.

In the absence of an iron enriched diet, Hfe-KO mice presented already an increase in serum iron concentration (+54.6%) and transferrin saturation (Trf Sat; +84.6%), when compared to normal levels observed into WT (Fig 1), reflecting the disruption of normal iron metabolism already present in KO mice as a consequence of the Hfe loss of function. Once fed with an iron enriched diet, these levels suffered a further increase in KO mice, but the most important being observed in the serum ferritin levels, which increased 503.2%, reflecting a likely increase in total iron accumulation in tissues. In WT controls, all three parameters analyzed increased also when the animals were given the iron enriched diet, approaching the levels observed in KO animals fed the same diet for circulating levels of iron and transferrin saturation, while their levels of serum ferritin increased but remained much lower than those of the KO+I group. Therefore, serum ferritin appeared to be the most sensitive parameter responding to iron overload under the experimental conditions used.

The levels of iron accumulation on the surface of bone trabeculae, evaluated by Perls staining, showed a clear difference between [C] and [KO] groups, with the latter presenting an increase in iron accumulation on the surface of trabeculae together with an increase of iron deposits in bone marrow (Fig 1D). The feeding of an iron-enriched diet to [C+I] and [KO+I] groups led to a further increase in iron accumulation in the bone marrow (Fig 1D). However, [KO+I] showed a thicker layer of iron deposits on bone surface and clearly increased areas of iron deposits embedded in bone matrix as compared to their C+I controls, being more evident than in the KO animals that did not feed on an iron enriched diet (Fig 1D).

### Molecular response to iron overload, oxidative stress and inflammation in bone tissue of mice subjected to iron-enriched diet

**Fig 2:**
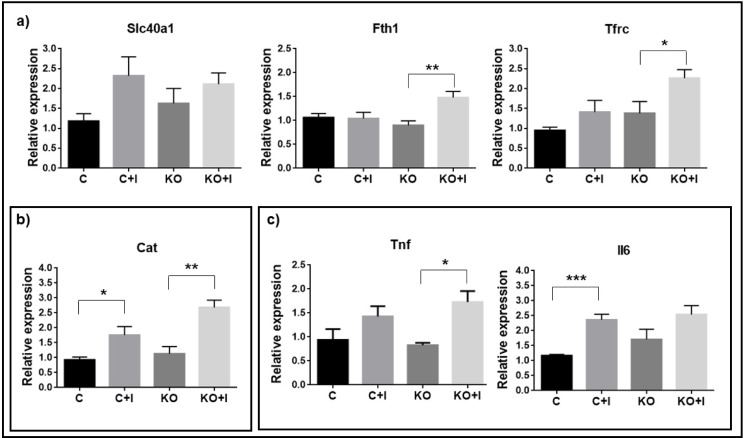
Gene expression analysis of iron metabolism, oxidative stress and inflammation markers in bone tissue. a) Iron metabolism: ferroportin (*Slc40a1*), ferritin heavy polypeptide 1 (*Fth1*) and transferrin receptor 1 (*Tfrc*). b) Oxidative stress: catalase (*Cat*). c) Inflammation response: Tumor necrosis factor (*Tnf*) and Interleukin 6 (*Il6*). Relative expression was obtained through ΔΔCt method and was normalized with *Gapdh*. Each group analyzed was composed by n = 6 animals for [C+I], [KO] and [KO+I], and by n = 5 for [C]. Statistical comparison was performed with T-test (Welch’s correction) between control and iron-enriched diets for both WT ([C] and [C+I]]) and Hfe-KO mice ([KO] and [KO+I])). Group deviations are expressed as standard error to the mean (SEM). Statistical significance: (*)-p<0.05; (**)-p<0.01; (***)-p<0.001.

Gene expression analysis of iron metabolism markers showed that the iron-enriched diet only promoted significant changes in Hfe-KO mice ([KO+I]), with increase in the expression of ferritin heavy polypeptide 1 (*Fth1*) and transferrin receptor-1 (*Tfrc*) (Fig 2A), while there were no significant changes in ferroportin (*Slc40a1*) expression.

The iron-enriched diet also promoted an increased expression of catalase (Cat) in both groups ([C+I] and [KO+I]) (Fig 2B). To determine the impact of iron-enriched diets on inflammation we evaluated the expression of tumor necrosis factor (*Tnf*) and interleukin 6 (*Il6*) in all groups. There appeared to be a consistent increase in both groups under the iron enriched diet, although only clearly significant for the expression of *Il6* in the WT and for the *Tnf* in the Hfe-KO mice (Fig 2C) suggesting that the dietary iron overload promoted inflammation.

### Iron-enriched diet accelerated bone loss in Hfe-KO mice

**Fig 3:**
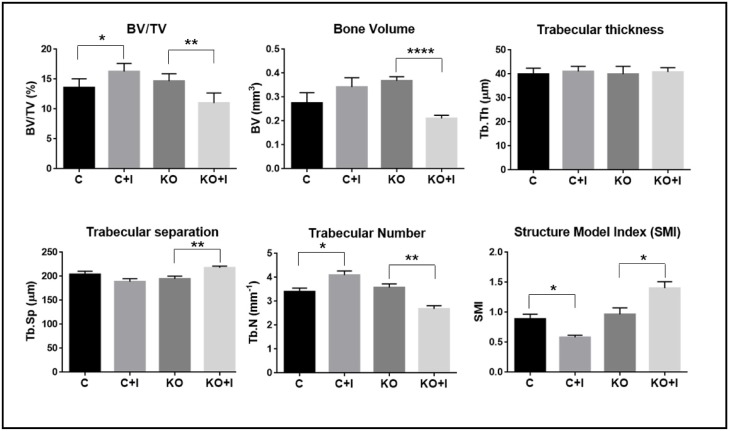
Evaluation of bone microarchitecture status by micro-Ct. Analysis of bone status was performed by determining microarchitecture parameters of bone volume fraction (BV/TV), bone volume (BV), trabecular thickness (Tb.Th), trabecular number (Tb.N), trabecular separation (Tb.Sp), and structural model index (SMI). Each group analyzed was composed by n = 6 animals for [C+I], [KO] and [KO+I], and n = 5 for [C]. Statistical comparison was performed with T-test (Welch’s correction) between control and iron-enriched diets for both WT ([C] and [C+I]]) and Hfe-KO mice ([KO] and [KO+I])). Group deviations are expressed as standard error to the mean (SEM). Statistical significance: (*)-p<0.05; (**)-p<0.01; (****)-p<0.0001.

Iron-enriched diet had different impacts on femur microarchitecture parameters for WT and Hfe-KO mice. In the [C+I] group there was an increase in BV/TV and Tb.N relatively to [C] which suggest an increase in trabecular bone (Fig 3). In contrast, the results from the [KO+I] group showed a decrease in BV, BV/TV and Tb.N when compared to [KO], while Tb.Sp and SMI were increased (Fig 3). These results were confirmed by the analysis of tibia micro-CT microarchitecture parameters and femur microarchitecture bidimensional analysis by Anilline blue staining.

**Fig 4:**
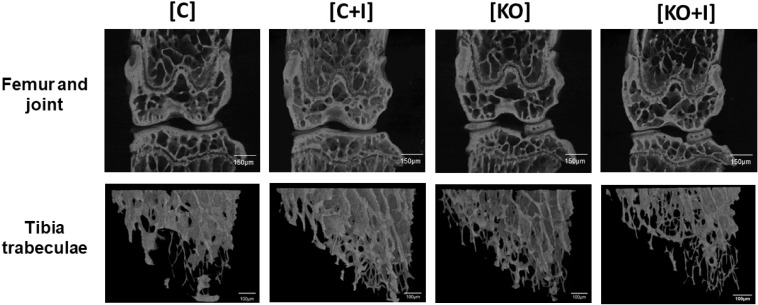
Micro-CT scans showing bone status from femur and tibia. Evaluation of microarchitecture parameters following three-dimensional reconstructions from micro-CT scans of femur and tibia as described previously. Three-dimensional reconstructions were obtained from femur and joint scans and from tibia trabecular bone scans using CTvox software (v3.1.1, Bruker, Belgium). Images scale bars equivalence: Femur and joint- 150μm, Tibia trabeculae- 100μm.

Representative images of bone microarchitecture for each group can be observed in tri-dimensional reconstructions of femur and tibia trabecular bone (Fig 4). This data suggests that, in contrast to WT mice, dietary iron supplementation has a deleterious effect on bone metabolism in Hfe-KO mice, in agreement with the microarchitecture parameters measured in the same animals.

### Iron-enriched diet contributes to decreased bone formation

**Fig 5:**
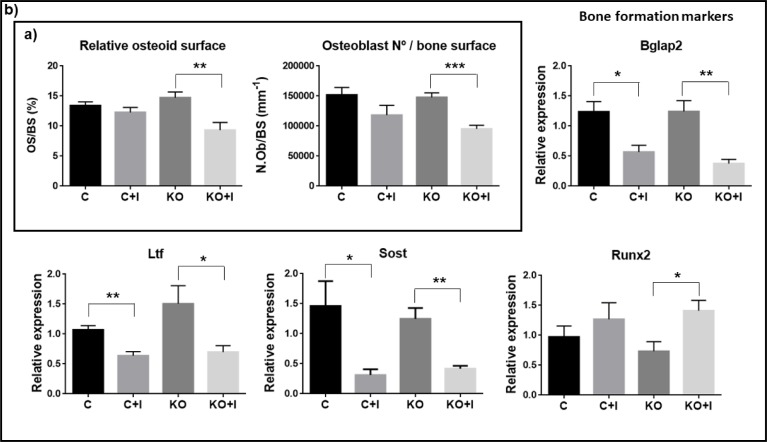
Impact of iron overload on bone formation. a) Evaluation of osteoid surface (OS/BS) and number of osteoblast (N.Ob/BS). b) Gene expression of bone formation markers: osteocalcin (*Bglap2*), lactoferrin (*Ltf*), sclerostin (*Sost*) and runt related transcription factor 2 (*Runx2*). Relative expression was obtained by ΔΔCt method and was normalized with *Gapdh*. Each group analyzed was composed by n = 6 animals for [C+I], [KO] and [KO+I], and by n = 5, for [C]. Statistical comparison was performed with T-test (Welch’s correction) between control and iron-enriched diets for both WT ([C] and [C+I]]) and Hfe-KO mice ([KO] and [KO+I])). Group deviations are expressed as standard error to the mean (SEM). Statistical significance: (*)-p<0.05; (**)-p<0.01; (***)-p<0.001.

To determine the impact of dietary iron supplementation on bone formation and mineralization, the relative osteoid surface (OS/BS) and number of osteoblasts per bone surface (N.Ob/BS) were evaluated, as well as the expression of markers associated with bone formation. The results show that both OS/BS and N.Ob/BS were statistically decreased in the Hfe-KO mice subjected to iron-enriched diet (Fig 5A), thus confirming a decrease in bone formation in this group, consistent with the results obtained by micro-CT analysis.

Analysis of the molecular markers for bone formation and mineralization showed a decrease for osteocalcin-2 (*Bglap2*), lactoferrin (*Ltf*) and sclerostin (*Sost*) expression in WT and Hfe-KO mice subjected to iron-enriched diets (Fig 5), consistent with a decrease in bone formation in the presence of iron overload. However, expression of runt related transcription factor 2 (*Runx2*), which is associated with early osteoblast differentiation, presented either no significant changes or only a moderate increase of expression in response to iron-enriched diet in both groups fed an iron enriched diet.

### Iron-enriched diet promotes a decrease in osteoclast number and activity preventing a deleterious effect on bone microarchitecture in WT mice

**Fig 6:**
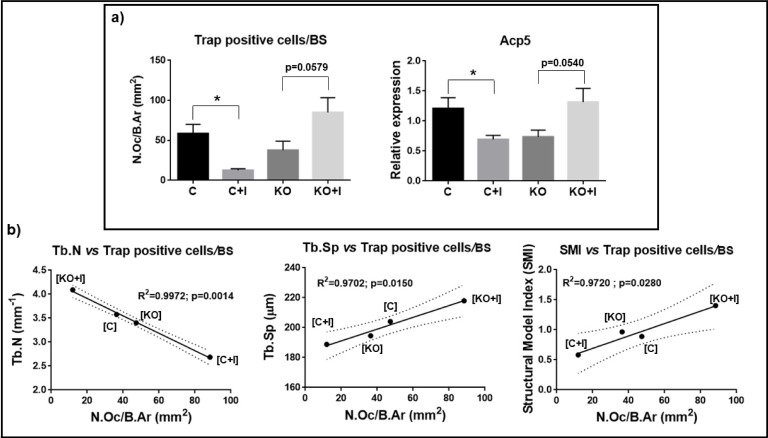
Trap positive cells per bone surface and correlation with trabecular bone microarchitecture. a) Number of trap positive cells per trabeculae surface and relative expression of acid phosphatase 5, tartrate resistant (*Acp5*) obtained by ΔΔCt method normalized with *Gapdh*. Each group analyzed was composed by n = 6 animals for [C+I], [KO] and [KO+I], and by n = 5, for [C]. b) Correlation between number of osteoclasts per trabeculae surface with Tb.N, Tb.Sp and SMI. Correlation was determined through Pearson’s test, with significance of p<0.05. Dashed line identifies confidence interval (95%). Statistical comparison with T-test (Welch’s correction) between control and iron-enriched diets for both WT ([C] and [C+I]]) and Hfe-KO mice ([KO] and [KO+I])). Group deviations are expressed as standard error to the mean (SEM). Statistical significance: (*)-p<0.05.

In the presence of iron-enriched diet, the number of Trap positive cells per bone surface was significantly decreased in WT animals ([C+I]), associated with a clear downregulation of the expression of tartrate-resistant acid phosphatase type 5 (*Acp5*) (Fig 6A). The iron overload-related decrease in number and activity of osteoclasts resulted in less resorption and thus an increase in bone mass, explaining the observed increase in BV/TV obtained from the micro-CT parameters. In contrast, both parameters appeared to be increased in the KO mice under an iron rich diet, despite being at the limit of significance, consistent once again with the micro-CT data

**Fig 7:**
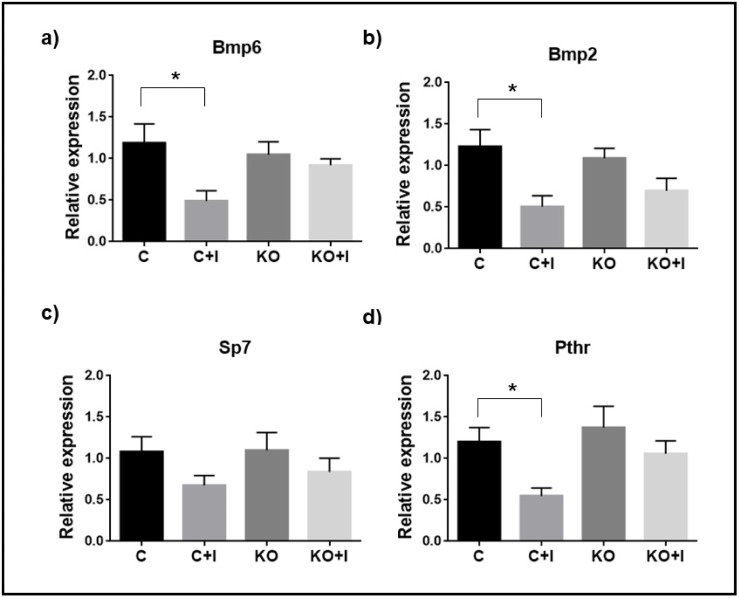
Gene expression of markers associated with regulation of bone metabolism. Relative expression: a) bone morphogenic protein 6 (*Bmp6*); b) Bone morphogenic protein 2 (*Bmp2*); c) Sp7 transcription factor 7 (*Sp7*); d) Parathyroid hormone 1 receptor (*Pthr*). Expression was determined by ΔΔCt method and normalized with *Gapdh*. Each group was composed by n = 6 animals ([C+I], [KO] and [KO+I]) except for [C] (n = 5). Comparison among groups was done by unpaired t-student test with Welch's correction and significance of p<0.05. Group deviations are expressed as standard error to the mean (SEM). Statistical significance: (*) = p<0.05.

Evaluation of the correlation between N.Oc/B.Ar relatively to Tb.N, Tb.Sp and SMI showed a statistical significant linear correlation between N.Oc/B.Ar and Tb.N (p<0.0014), Tb.Sp (p<0.0150) and SMI (p<0.0280), with negative correlation for Tb.N and positive correlation to Tb.Sp and SMI, (Fig 6B). The correlation between osteoclast number/bone surface and bone microarchitecture parameters is consistent with the phenotypes observed in WT and Hfe-KO mice fed with the iron-enriched diets. In addition, [C+I] mice revealed significant downregulation of bone morphogenetic proteins 2 (*Bmp2*) and 6 (*Bmp6*) and parathyroid hormone 1 receptor (*Pthr*) relatively to [C] (Fig 7), which was not observed in Hfe-KO subject to iron rich diet (Fig 7). Altogether the results obtained are in agreement with a repression of osteoclastogenesis in [C+I] but not in [KO+I] mice.

## Discussion

To study the effects of dietary iron supplementation on bone metabolism and which molecular mechanisms could be involved on the onset and progression of OP phenotype, we have used an HH biological model for systemic iron overload, the Hfe-KO mouse [14], and evaluated its bone status when subjected to two different diets, standard (200 mg/Kg of Fe) and iron-enriched (200mg/Kg of Fe plus 1% iron carbonyl relatively to total ratio weight). In a parallel experiment a similar dietary treatment was given to WT mice to evaluate their response to iron overload in the absence of Hfe mutations. The iron-enriched diet led to an osteoporotic phenotype only on the Hfe-KO animals while in WT mice it led to an increase in BV/TV most likely by a repression of osteoclastogenesis, not observed in the Hfe-KO.

The dietary iron supplementation caused systemic iron overload on both WT and Hfe-KO mice, as described previously [22–25]. [C+I] and [KO+I] mice also showed abnormal elevation of serum ferritin concentrations (Fig 1C), which suggested a significant increase in total body iron stores associated with signs of inflammation, as described for HH patients [26] and also observed in similar studies associated with iron carbonyl supplementation [24]. In addition, increased iron accumulation in trabecular bone was observed for [KO], [C+I] and [KO+I] mice, in agreement with previous works [25,27]. In [C] group, iron deposits were only detected on bone marrow (Fig 1D) but following dietary iron overload ([C+I]), iron deposits were also found in bone marrow and skeletal tissue, but mainly in the osteoid. In contrast, in [KO] group a severe iron accumulation was already observed even in the absence of dietary iron overload, because of the systemic increase in iron due to Hfe mutation, with iron deposits already present on bone surface as well as in osteoid (Fig -d). These results were even more evident in the [KO+I] group, with iron deposits also found within bone mineralized matrix (Fig 1D) indicating that Hfe-KO mice are more susceptible to iron overload, a result also in agreement with our previous data (Fig A in S1 File.).

Bone loss was accelerated in [KO+I], with significant decrease on bone formation parameters such as relative osteoid surface and osteoblasts number per bone surface (Fig 5A). In addition, bone formation markers like *Bglap2*, *Ltf* and *Sost* were downregulated (Fig 5B), in agreement with the histomorphometric results observed in the same mice (Fig 3), thus confirming a negative effect of the iron-enriched diet in bone metabolism. These results are also in agreement with previous studies [25,28,29]. Together, these results suggested that osteoblast death and mineralization impairment could represent also inhibition of osteoblast differentiation as described *in vitro* previously [30]. However *Runx2* was upregulated in [KO+I] group which could be explained by the significant downregulation of *Sost* (Fig 5B), which is known to inhibit WNT signaling by interacting with Low density lipoprotein receptor-related protein 5 (LRP5) [31]. And attending that WNT signaling is important for RUNX2 induction [32,33], results obtained suggest that downregulation of *Sost* could lead to an increase in WNT pathway and thus favor *Runx2* upregulation in the context of this experiment. Furthermore, those results suggest a mechanism to compensate bone loss promoted by iron overload which can lead to osteoblast differentiation and recruiting. Nevertheless, this is not dismissive of the possible negative impact of iron overload on osteoblast differentiation as shown by significant downregulation of *Bglap2* and decrease in osteoblast number, which could be the result of the associated iron toxicity on mature and immature osteoblasts and osteocytes. Altogether, these results suggest the uncoupling between bone formation and resorption mechanisms.

The abovementioned uncoupling between bone formation and resorption was only evident in mice Hfe KO subject to iron rich diet and the analysis of iron metabolism markers revealed the impact of the conjugation between enriched diet and Hfe loss of function, resulting in an upregulation of *Fth1* and *Tfrc* in the [KO+I] group (Fig 2A). Increased expression of these genes has been associated with bone loss phenotypes, with ferritin ferroxidase activity previously shown to inhibit calcium deposition and osteocalcin expression [30,34], while upregulation of *Tfrc*, previously observed in bone tissue, was suggested to facilitate iron accumulation associated with bone loss [28]. *Fth1* and *Tfrc* have been shown to be regulated by increased iron concentrations at post-transcription level by the presence of iron response elements (IREs) which can be recognized by Iron response proteins (IRPs) 1 and 2 [28]. Usually, upon high iron concentrations, *Fht1* mRNA expression is not affected but translation is increased while translation of *Tfrc* is decreased due to mRNA destabilization by absence of IRP’s availability for binding to its 3’ UTR IRE’s due to competition with high intracellular iron concentrations [28]. In contrast, if a ferroptosis mechanism is triggered, this can lead to the upregulation of both *Fth1* [35] and *Tfrc* [36] in order to induce cell death. Ferroptosis is a mechanism of programmed cell death which uses iron as cofactor when cells cannot regulate high levels of oxidative stress [37,38]. Indeed, *Fth1* can be also upregulated by increased levels of oxidative stress in order to decrease intracellular iron labile concentrations and avoid formation of ROS [39], to which we can add the upregulation of *Cat* and *Tnf* (Fig 2C), already known to be increased in response to oxidative stress [40].

Since [KO+I] group showed upregulation of *Cat* and *Tnf* (Fig 2C), which also favors osteoclastogenesis [41–43], this contributes to explain the uncoupling of bone metabolism (i) by the increase of osteoclast activity, as shown by an increase in *Acp5* expression and number of Trap positive cells relatively to [KO] group (Fig 6A), and (ii) by promoting osteoblast death [44], as shown by the decrease in osteoblast number detected (Fig 5A). Together, these evidences suggest that ferroptosis could be a mechanism associated to bone loss in [KO+I]. In addition, the absence of statistically significant changes in *Slc40a1* expression may reflect the inability of bone cells to export iron to alleviate intracellular iron toxicity.

Evidences of iron overload conditions were also observed in [C+I] group (Fig 1), however there were no significant changes in *Fth1* and *Tfrc* expression (Fig 2), as well as no significant changes in the number of osteoblasts in their bones indicating that, despite the fact that [C+I] showed some evidences of bone loss, as shown by *Bglap2*, *Ltf* and *Sost* downregulation (Fig 5B), iron metabolism markers were unchanged (Fig 2A), even in the presence of iron overload conditions (Figs 1 and 2). In fact, it was possible to observe increases in bone volume fraction, trabecular number and trabecular organization in the [C+I] group, which was unexpected given the systemic iron overload. The main difference observed between [C+I] and [KO+I] groups was the maintenance of bone metabolism coupling in [C+I], which was lost for [KO+I] group. In [C+I] mice, and in clear contrast with [KO+I] mice, bone resorption parameters were significantly downregulated, with a strong decrease in Trap positive cells and downregulation of *Acp5* expression. Although these mice also showed a downregulation of bone formation markers, our results suggest that the decrease in bone resorption was the main reason for the increase in bone volume fraction and number of trabeculae detected in [C+I].

The causal mechanism for decreased osteoclast activity in [C+I] could be related with impairment of osteocyte/osteoclast communication, due probably to increase osteocyte death as evidenced by downregulation of *Sost* expression, known to affect osteoclast recruitment and thus responsible for a downregulation of PTH/PTH1R signaling [44], in agreement with the observed downregulation of *Pth1r* in the [C+I] mice (Fig 7). In addition, downregulation of *Bmp6* and *Bmp2* in response to iron overload, similar to previously observed under inflammation conditions [45] (Fig 7), suggests an inhibition of BMP signaling which, together with the downregulation of *Sost*, can favor canonical WNT pathway upregulation [46] and consequently negatively affect osteoclasteogenesis [47]. These results were similar to those described for Bmpr1-KO mouse, in which inhibition of BMP signaling and repressed expression of *Sost* negatively affected osteoclastogenesis resulting in increased bone formation [46]. In addition, it has been shown that Trap activity can be inhibited by increased iron exposition *in vitro* [48]. Altogether, these results could contribute to explain the variability associated with bone loss phenotype in HH patients. Indeed, the systemic nature of iron overload associated with Hfe loss of function can affect primarily mature osteoclast activity until iron toxicity can promote generalized osteoblast death because of the development of iron toxicity and increased oxidative stress. This could in turn lead to an increase in osteoclastogenesis to promote bone remodeling to substitute affected bone, consistent with what was observed in the [KO+I] group.

The iron-enriched diet induced an inflammatory response, as observed by the upregulation of inflammatory markers like *Il6* and *Tnf*, consistent with the induction of an osteoporotic phenotype [41,43,49,50]. Furthermore, since the levels of inflammation are already increased in the Hfe-KO mice [45], this could explain why the further increase resulting from iron overload is not significant in the [KO+I] as shown in Fig 2C.

In conclusion, our results indicate that dietary factors associated with iron overload in the presence of HFE loss of function can promote oxidative stress and inflammation, which can be crucial for the development of the HH-related osteoporosis.

## Supporting information

S1 File **Table A. Primers sequences for gene expression analysis**. Sequences and references of primers used in gene expression analysis by qPCR. **Fig A Microarchitecture parameters between WT and Hfe-KO mice (12 months old).** a- Bone microarchitecture parameters evaluated were bone volume fraction (BV/TV), bone volume (BV), trabecular thickness (Tb.Th), trabecular number (Tb.N), trabecular separation (Tb.Sp), and structural model index (SMI). WT and Hfe-KO groups were composed each by n = 8 mice and comparison between WT and Hfe-KO conditions was made with unpaired t-student test with Welch's correction and significance of p<0.05. Group deviations are expressed as confidence interval at 95%. (*). Indication of statistical significance when compared with WT group. b- Three-dimensional reconstructions represented were from tibia trabecular bone of WT and Hfe-KO mice (12months) and were done with CTvox software (v3.1.1, Bruker, Belgium). **Material and methods section A. Bone status of Hfe-KO mouse (12 months old). Material and methods section B. Micro-CT scan (WT and Hfe-KO, 12 months old).**
(DOCX)

## References

[1] Panwar B, Gutiérrez OM. Disorders of Iron Metabolism and Anemia in Chronic Kidney Disease. Semin Nephrol. Elsevier; 2016;36: 252–261. 10.1016/j.semnephrol.2016.05.002 27475656 10.1016/j.semnephrol.2016.05.002

[2] Chen B, Li G, Shen Y, Huang X, Xu Y. Reducing iron accumulation: A potential approach for the prevention and treatment of postmenopausal osteoporosis. Exp Ther Med. 2015;10: 7–11. 10.3892/etm.2015.2484 26170904 10.3892/etm.2015.2484 PMC4486897

[3] Brissot P, Loréal O, Loreal O. Iron metabolism and related genetic diseases: a cleared land, keeping mysteries. J Hepatol. 2015;64: 505–515. 10.1016/j.jhep.2015.11.009 26596411 10.1016/j.jhep.2015.11.009

[4] Pietrangelo A. Hereditary hemochromatosis: Pathogenesis, diagnosis, and treatment. Gastroenterology. 2010;139: 393–408. 10.1053/j.gastro.2010.06.013 20542038 10.1053/j.gastro.2010.06.013

[5] Porto G, Brissot P, Swinkels DW, Zoller H, Kamarainen O, Patton S, et al EMQN best practice guidelines for the molecular genetic diagnosis of hereditary hemochromatosis (HH). Eur J Hum Genet. 2016;24: 479–95. 10.1038/ejhg.2015.128 26153218 10.1038/ejhg.2015.128 PMC4929861

[6] Merryweather-Clarke AT, Pointon JJ, Shearman JD, Robson KJ. Global prevalence of putative haemochromatosis mutations. J Med Genet. 1997;34: 275–278. 10.1136/jmg.34.4.275 9138148 10.1136/jmg.34.4.275 PMC1050911

[7] Jeney V. Clinical Impact and Cellular Mechanisms of Iron Overload-Associated Bone Loss. Front Pharmacol. 2017;8: 1–11. 10.3389/fphar.2017.00001 28270766 10.3389/fphar.2017.00077 PMC5318432

[8] Cheng Q, Zhang X, Jiang J, Zhao G, Wang Y, Xu Y, et al Postmenopausal Iron Overload Exacerbated Bone Loss by Promoting the Degradation of Type I Collagen. Biomed Res Int. Hindawi; 2017;2017: 1–9. 10.1155/2017/1345193 28620614 10.1155/2017/1345193 PMC5460413

[9] Valenti L, Varenna M, Fracanzani a. L, Rossi V, Fargion S, Sinigaglia L. Association between iron overload and osteoporosis in patients with hereditary hemochromatosis. Osteoporos Int. 2009;20: 549–555. 10.1007/s00198-008-0701-4 18661088 10.1007/s00198-008-0701-4

[10] Guggenbuhl P, Deugnier Y, Boisdet JF, Rolland Y, Perdriger a., Pawlotsky Y, et al Bone mineral density in men with genetic hemochromatosis and HFE gene mutation. Osteoporos Int. 2005;16: 1809–1814. 10.1007/s00198-005-1934-0 15928800 10.1007/s00198-005-1934-0

[11] Chan CKY, Mason A, Cooper C, Dennison E. Novel advances in the treatment of osteoporosis. Br Med Bull. 2016; 1–13. 10.1093/bmb/ldw008 27558130 10.1093/bmb/ldw033 PMC5027910

[12] Okyay E, Ertugrul C, Acar B, Sisman AR, Onvural B, Ozaksoy D. Comparative evaluation of serum levels of main minerals and postmenopausal osteoporosis. Maturitas. 2013;76: 320–5. 10.1016/j.maturitas.2013.07.015 24011991 10.1016/j.maturitas.2013.07.015

[13] Abraham R, Walton J, Russell L, Wolman R, Wardley-Smith B, Green JR, et al Dietary determinants of post-menopausal bone loss at the lumbar spine: A possible beneficial effect of iron. Osteoporos Int. 2006;17: 1165–1173. 10.1007/s00198-005-0033-6 16758136 10.1007/s00198-005-0033-6

[14] Bahram S, Gilfillan S, Kühn LC, Moret R, Schulze JB, Lebeau a, et al Experimental hemochromatosis due to MHC class I HFE deficiency: immune status and iron metabolism. Proc Natl Acad Sci U S A. 1999;96: 13312–13317. 10.1073/pnas.96.23.13312 10557317 10.1073/pnas.96.23.13312 PMC23944

[15] Feder JN, Gnirke A, Thomas W, Tsuchihashi Z, Ruddy DA, Basava A, et al A novel MHC class I-like gene is mutaded in patients with hereditary haemochromatosis. Nat Genet. 1996;14: 353–6. 10.1038/ng1196-353 8696333 10.1038/ng0896-399

[16] Camacho A, Simão M, Ea H-KH, Cohen-Solal M, Richette P, Branco J, et al Iron overload in a murine model of hereditary hemochromatosis is associated with accelerated progression of osteoarthritis under mechanical stress. Osteoarthr Cartil. 2016;24: 494–502. 10.1016/j.joca.2015.09.007 26403062 10.1016/j.joca.2015.09.007

[17] Bouxsein ML, Boyd SK, Christiansen BA, Guldberg RE, Jepsen KJ, Müller R. Guidelines for assessment of bone microstructure in rodents using micro-computed tomography. J Bone Miner Res. 2010;25: 1468–1486. 10.1002/jbmr.141 20533309 10.1002/jbmr.141

[18] Hildebrand T, Rüegsegger P. Quantification of Bone Microarchitecture with the Structure Model Index. Comput Methods Biomech Biomed Engin. 1997;1: 15–23. 10.1080/01495739708936692 11264794 10.1080/01495739708936692

[19] Heusschen R, Muller J, Binsfeld M, Marty C, Plougonven E, Dubois S, et al SRC kinase inhibition with saracatinib limits the development of osteolytic bone disease in multiple myeloma. Oncotarget. 2014;7 doi: 10.18632/oncotarget.8750 27095574 10.18632/oncotarget.8750 PMC5058712

[20] Saintier D, Khanine V, Uzan B, Ea HK, de Vernejoul MC, Cohen-Solal ME. Estradiol inhibits adhesion and promotes apoptosis in murine osteoclasts in vitro. J Steroid Biochem Mol Biol. 2006;99: 165–173. 10.1016/j.jsbmb.2006.01.009 16621521 10.1016/j.jsbmb.2006.01.009

[21] Pfaffl MW. A new mathematical model for relative quantification in real-time RT-PCR. 2001;29: 16–21. 10.1093/nar/29.9.e45 10.1093/nar/29.9.e45 PMC55695 11328886

[22] Chua ACG, Olynyk JK, Leedman PJ, Trinder D. Nontransferrin-bound iron uptake by hepatocytes is increased in the Hfe knockout mouse model of hereditary hemochromatosis. Blood. 2004;104: 1519–1525. 10.1182/blood-2003-11-3872 15155457 10.1182/blood-2003-11-3872

[23] Trinder D, Olynyk JK, Sly WS, Morgan EH. Iron uptake from plasma transferrin by the duodenum is impaired in the Hfe knockout mouse. Proc Natl Acad Sci U S A. 2002;99: 5622–6. 10.1073/pnas.082112299 11943867 10.1073/pnas.082112299 PMC122820

[24] Choi JS, Koh I-U, Lee HJ, Kim WH, Song J. Effects of excess dietary iron and fat on glucose and lipid metabolism. J Nutr Biochem. Elsevier Inc.; 2013;24: 1634–1644. 10.1016/j.jnutbio.2013.02.004 23643521 10.1016/j.jnutbio.2013.02.004

[25] Tsay J, Yang Z, Ross FP, Cunningham-Rundles S, Lin H, Coleman R, et al Bone loss caused by iron overload in a murine model: importance of oxidative stress. Blood. 2010;116: 2582–2589. 10.1182/blood-2009-12-260083 20554970 10.1182/blood-2009-12-260083 PMC2953890

[26] Crownover BK, Covey CJ. Hereditary hemochromatosis. Am Fam Physician. 2013;87: 183–190. 10.1179/1024533213Z.000000000222 23418762

[27] Guggenbuhl P, Fergelot P, Doyard M, Libouban H, Roth M-P, Gallois Y, et al Bone status in a mouse model of genetic hemochromatosis. Osteoporos Int. 2011;22: 2313–9. 10.1007/s00198-010-1456-2 20976594 10.1007/s00198-010-1456-2

[28] Xu Z, Sun W, Li YY, Ling S, Zhao C, Zhong G, et al The regulation of iron metabolism by hepcidin contributes to unloading-induced bone loss. Bone. 2016;94: 152–161. 10.1016/j.bone.2016.09.023 27686598 10.1016/j.bone.2016.09.023

[29] Doyard M, Chappard D, Leroyer P, Roth MP, Loréal O, Guggenbuhl P. Decreased bone formation explains osteoporosis in a genetic mouse model of hemochromatosiss. PLoS One. 2016;11: 1–10. 10.1371/journal.pone.0148292 26829642 10.1371/journal.pone.0148292 PMC4734777

[30] Balogh E, Tolnai E, Nagy B, Nagy B, Balla G, Balla J, et al Iron overload inhibits osteogenic commitment and differentiation of mesenchymal stem cells via the induction of ferritin. Biochim Biophys Acta—Mol Basis Dis. 2016;1862: 1640–1649. 10.1016/j.bbadis.2016.06.003 27287253 10.1016/j.bbadis.2016.06.003

[31] Balemans W, Piters E, Cleiren E, Ai M, Van Wesenbeeck L, Warman ML, et al The binding between sclerostin and LRP5 is altered by DKK1 and by high-bone mass LRP5 mutations. Calcif Tissue Int. 2008;82: 445–453. 10.1007/s00223-008-9130-9 18521528 10.1007/s00223-008-9130-9

[32] Mbalaviele G, Sheikh S, Stains JP, Salazar VS, Cheng SL, Chen D, et al β-catenin and BMP-2 synergize to promote osteoblast differentiation and new bone formation. J Cell Biochem. 2005;94: 403–418. 10.1002/jcb.20253 15526274 10.1002/jcb.20253 PMC2647989

[33] Gaur T, Lengner CJ, Hovhannisyan H, Bhat RA, Bodine PVN, Komm BS, et al Canonical WNT signaling promotes osteogenesis by directly stimulating Runx2 gene expression. J Biol Chem. 2005;280: 33132–33140. 10.1074/jbc.M500608200 16043491 10.1074/jbc.M500608200

[34] Zarjou A, Jeney V, Arosio P, Poli M, Zavaczki E, Balla G, et al Ferritin ferroxidase activity: a potent inhibitor of osteogenesis. J Bone Miner Res. 2010;25: 164–172. 10.1359/jbmr.091002 19821764 10.1359/jbmr.091002

[35] Gao M, Monian P, Pan Q, Zhang W, Xiang J, Jiang X. Ferroptosis is an autophagic cell death process. Cell Res. Nature Publishing Group; 2016;26: 1021–1032. 10.1038/cr.2016.95 27514700 10.1038/cr.2016.95 PMC5034113

[36] Totsuka K, Ueta T, Uchida T, Roggia MF, Nakagawa S, Vavvas DG, et al Oxidative stress induces ferroptotic cell death in retinal pigment epithelial cells. Exp Eye Res. Elsevier; 2018; 1–9. 10.1016/j.exer.2018.08.019 30171859 10.1016/j.exer.2018.08.019 PMC7418497

[37] Gao M, Monian P, Quadri N, Ramasamy R, Jiang X. Glutaminolysis and Transferrin Regulate Ferroptosis. Mol Cell. 2015;59: 298–308. 10.1016/j.molcel.2015.06.011 26166707 10.1016/j.molcel.2015.06.011 PMC4506736

[38] Cao JY, Dixon SJ. Mechanisms of ferroptosis. Cell Mol Life Sci. Springer International Publishing; 2016; 10.1007/s00018-016-2194-1 27048822 10.1007/s00018-016-2194-1 PMC4887533

[39] Torti FM, Torti S V. Regulation of ferritin genes and protein. Blood. 2002;99: 3505–3516. 10.1182/blood.V99.10.3505 11986201 10.1182/blood.v99.10.3505

[40] Ahmed SMU, Luo L, Namani A, Wang XJ, Tang X. Nrf2 signaling pathway: Pivotal roles in inflammation. Biochim Biophys Acta—Mol Basis Dis. Elsevier B.V.; 2017;1863: 585–597. 10.1016/j.bbadis.2016.11.005 27825853 10.1016/j.bbadis.2016.11.005

[41] Osta B, Benedetti G, Miossec P. Classical and paradoxical effects of TNF-alpha on bone homeostasis. Front Immunol. 2014;5: 1–9. 10.3389/fimmu.2014.00001 24592264 10.3389/fimmu.2014.00048 PMC3923157

[42] Lam J, Takeshita S, Barker JE, Kanagawa O, Ross FP, Teitelbaum SL. TNF-α induces osteoclastogenesis by direct stimulation of macrophages exposed to permissive levels of RANK ligand. J Clin Invest. 2000;106: 1481–1488. 10.1172/JCI11176 11120755 10.1172/JCI11176 PMC387259

[43] Wei S, Kitaura H, Zhou P, Patrick Ross F, Teitelbaum SL. IL-1 mediates TNF-induced osteoclastogenesis. J Clin Invest. 2005;115: 282–290. 10.1172/JCI23394 15668736 10.1172/JCI23394 PMC544608

[44] Zheng L, Wang W, Ni J, Mao X, Song D, Liu T, et al Role of autophagy in tumor necrosis factor-α-induced apoptosis of osteoblast cells. J Investig Med. 2017;65: 1014–1020. 10.1136/jim-2017-000426 28634253 10.1136/jim-2017-000426 PMC5537511

[45] Salama MF, Bayele HK, Srai SSK. Tumour necrosis factor alpha downregulates human hemojuvelin expression via a novel response element within its promoter. J Biomed Sci. Journal of Biomedical Science; 2012;19: 1 10.1186/1423-0127-19-1 22998440 10.1186/1423-0127-19-83 PMC3500654

[46] Kamiya N, Ye L, Kobayashi T, Mochida Y, Yamauchi M, Kronenberg HM, et al BMP signaling negatively regulates bone mass through sclerostin by inhibiting the canonical Wnt pathway. Development. 2008;135: 3801–3811. 10.1242/dev.025825 18927151 10.1242/dev.025825 PMC2694443

[47] Spencer GJ, Utting JC, Etheridge SL, Arnett TR, Genever PG. Wnt signalling in osteoblasts regulates expression of the receptor activator of NFkappaB ligand and inhibits osteoclastogenesis in vitro. J Cell Sci. 2006;119: 1283–1296. 10.1242/jcs.02883 16522681 10.1242/jcs.02883

[48] Xie W, Lorenz S, Dolder S, Hofstetter W. Extracellular Iron is a Modulator of the Differentiation of Osteoclast Lineage Cells. Calcif Tissue Int. Springer US; 2016;98: 275–283. 10.1007/s00223-015-0087-1 26615413 10.1007/s00223-015-0087-1

[49] Li X, Zhou ZY, Zhang YY, Yang HL. IL-6 contributes to the defective osteogenesis of bone marrow stromal cells from the vertebral body of the glucocorticoid-induced osteoporotic mouse. PLoS One. 2016;11: 1–19. 10.1371/journal.pone.0154677 27128729 10.1371/journal.pone.0154677 PMC4851291

[50] Dai J, Lin D, Zhang J, Habib P, Smith P, Murtha J, et al Chronic alcohol ingestion induces osteoclastogenesis and bone loss through IL-6 in mice. J Clin Invest. 2000;106: 887–895. 10.1172/JCI10483 11018077 10.1172/JCI10483 PMC381425

