# Correction: Iron-enriched diet contributes to early onset of osteoporotic phenotype in a mouse model of hereditary hemochromatosis

**DOI:** 10.1371/journal.pone.0216377

**Published:** 2019-04-29

**Authors:** Márcio Simão, António Camacho, Agnès Ostertag, Martine Cohen-Solal, I. Jorge Pinto, Graça Porto, Hang-Korng Ea, M. Leonor Cancela

The seventh author's name is spelled incorrectly. The correct name is: Hang-Korng Ea.

There are errors in the author affiliations. The correct affiliations are as follows: Márcio Simão^1,2,3^, António Camacho^4^, Agnès Ostertag^5^, Martine Cohen-Solal^5^, I. Jorge Pinto^6^, Graça Porto^6,7,8^, Hang-Korng Ea^5^, M. Leonor Cancela^2,3,9^.

**1** PhD Program in Biomedical Sciences, Department of Biomedical Sciences and Medicine (DCBM), University of Algarve, Faro, Portugal, **2** Centre of Marine Sciences (CCMAR), University of Algarve, Faro, Portugal, **3** Department of Biomedical Sciences and Medicine (DCBM), University of Algarve, Faro, Portugal, **4** Department of Orthopedics, Hospital de Cascais, Alcabideche, Portugal, **5** Inserm U1132/ BIOSCAR, and Université Paris 7 Denis Diderot, Hôpital Lariboisière, Paris, France, **6** Basic and Clinical Research on Iron Biology, Institute for Molecular and Cell Biology (IBMC) and I3S –Instituto de Investigação e Inovação em Saúde, University of Porto, Porto, Portugal, **7** Pathology and Molecular Immunology Department, Institute of Biomedical Sciences Abel Salazar (ICBAS), University of Porto, Porto, Portugal, **8** Hematology Service, Hospital de Santo António, Centro Hospitalar do Porto, Porto, Portugal, **9** Algarve Biomedical Center, University of Algarve, Campus de Gambelas, 8005–139, Faro, Portugal.
