# Supplementary material for: Iron-enriched diet contributes to early onset of osteoporotic phenotype in a mouse model of hereditary hemochromatosis
Source: PLoS One. 2018 Nov 14;13(11):e0207441. doi: 10.1371/journal.pone.0207441 (PMC6241130; doi:10.1371/journal.pone.0207441)
Supplement: S1 File — Table A. Primers sequences for gene expression analysis. Sequences and references of primers used in gene expression analysis by qPCR. Fig A Microarchitecture parameters between WT and Hfe-KO mice (12 months old). a- Bone microarchitecture parameters evaluated were bone volume fraction (BV/TV), bone volume (BV), trabecular thickness (Tb.Th), trabecular number (Tb.N), trabecular separation (Tb.Sp), and structural model index (SMI). WT and Hfe-KO groups were composed each by n = 8 mice and comparison between WT and Hfe-KO conditions was made with unpaired t-student test with Welch's correction and significance of p<0.05. Group deviations are expressed as confidence interval at 95%. (*). Indication of statistical significance when compared with WT group. b- Three-dimensional reconstructions represented were from tibia trabecular bone of WT and Hfe-KO mice (12months) and were done with CTvox software (v3.1.1, Bruker, Belgium). Material and methods section A. Bone status of Hfe-KO mouse (12 months old). Material and methods section B. Micro-CT scan (WT and Hfe-KO, 12 months old). (DOCX) [file pone.0207441.s001.docx]

**Table A**- Primer sequences for gene expression analysis. Sequences and references of primers used in gene expression analysis by qPCR are indicated


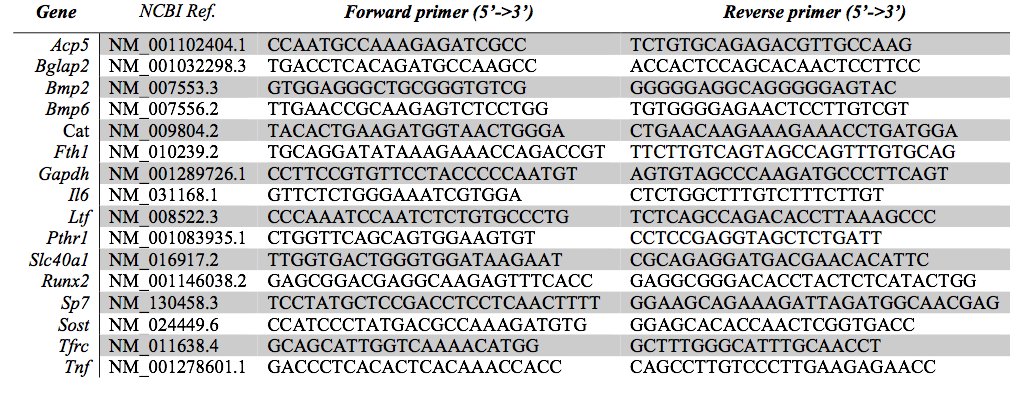


**Supplementary figures**


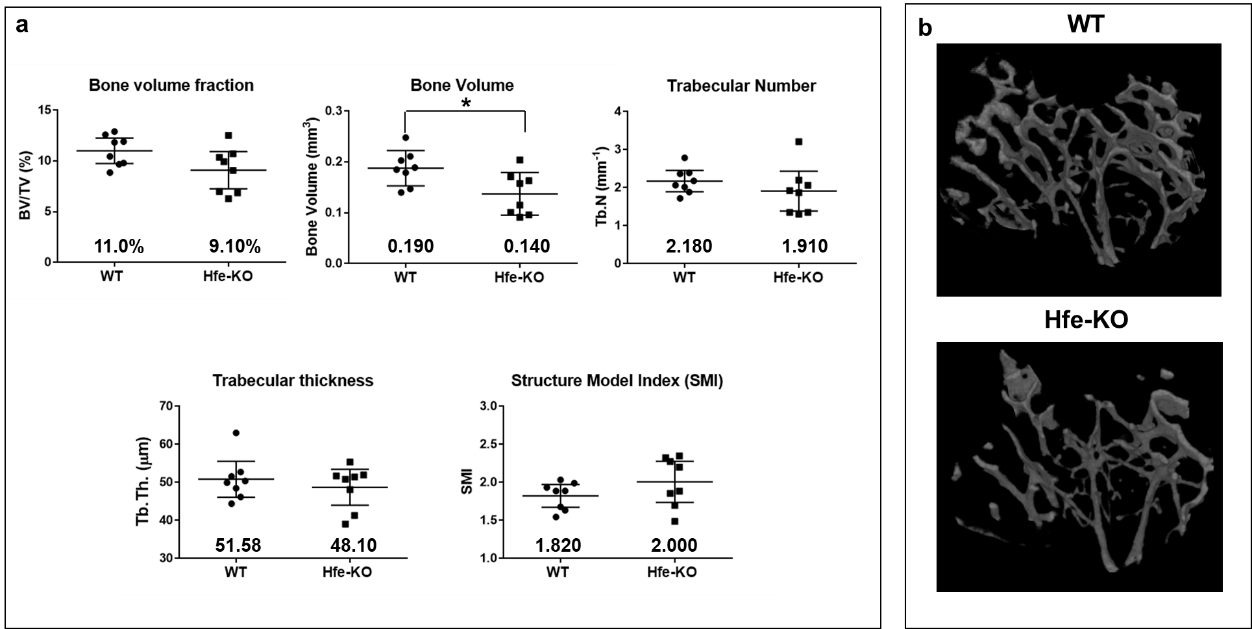


Fig.A- **Microarchitecture parameters between WT and Hfe-KO mice (12 months old).** a- Bone microarchitecture parameters evaluated were bone volume fraction (BV/TV), bone volume (BV), trabecular thickness (Tb.Th), trabecular number (Tb.N), trabecular separation (Tb.Sp), and structural model index (SMI). WT and Hfe-KO groups were composed each by n=8 mice and comparison between WT and Hfe-KO conditions was made with unpaired t-student test with Welch's correction and significance of p<0.05. Group deviations are expressed as confidence interval at 95%. (*). Indication of statistical significance when compared with WT group. b- Three-dimensional reconstructions represented were from tibia trabecular bone of WT and Hfe-KO mice (12months) and were done with CTvox software (v3.1.1, Bruker, Belgium).

**Supplementary material and methods**

**Material and methods section A. Bone status of Hfe-KO mouse (12 months old)**

In order to evaluate bone microarchitecture status of Hfe-KO mice with 12 months of age, two groups of mice, Hfe-KO mice (n=8) and C57BL/6 (n=8, used as control) were maintained at University of Algarve Animal Facility. Animals were kept in a 12hrs light/dark cycle, had access to water and food (SDS RM3A, iron content 161 mg/kg) *ad-libitum* from weaning up to 12 months of age until euthanasia and were maintained in specific pathogen-free conditions in individually ventilated cages. At sacrifice, right and left hindlimbs were collected as previously described in methods.

**Material and methods section B. Micro-CT scan (WT and Hfe-KO, 12 months old)**

Collection and preparation of samples for this analysis were done as previously described in methods. For acquisition, we used a Skyscan1172 device and images from each sample were acquired with a resolution of 2K pixels, with 8W power source, 70Kv voltage, using 0.5mm Al (Aluminium) filter, with 500ms exposure time, 0.4º step rotation and acquiring images in which voxel size were 5μm^3^. Datasets were then reconstructed by NRecon v1.6.9.8 software (Bruker, Belgium) and processed as described previously in methods for evaluation of bone microarchitecture status.
